# Diagnosis of invasive aspergillus tracheobronchitis facilitated by endobronchial ultrasound-guided transbronchial needle aspiration: a case report

**DOI:** 10.1186/1752-1947-3-9290

**Published:** 2009-11-23

**Authors:** Roberto F Casal, Roberto Adachi, Carlos A Jimenez, Mona Sarkiss, Rodolfo C Morice, Georgie A Eapen

**Affiliations:** 1 Department of Pulmonary Medicine, The University of Texas M. D. Anderson Cancer Center, Houston, Texas, USA; 2 Department of Anesthesiology and Perioperative Medicine, The University of Texas M. D. Anderson Cancer Center, Houston, Texas, USA

## Abstract

**Introduction:**

Invasive pulmonary aspergillosis is the most common form of infection by *Aspergillus species *among immunocompromised patients. Although this infection frequently involves the lung parenchyma, it is unusual to find it limited to the tracheobronchial tree, a condition known as invasive aspergillus tracheobronchitis.

**Case presentation:**

A 65 year-old Hispanic man from Bolivia with a history of chronic lymphocytic leukemia developed cough and malaise eight months after having an allogenic stem cell transplant. A computed tomography of the chest revealed an area of diffuse soft tissue thickening around the left main stem bronchus, which was intensely fluorodeoxyglucose-avid on positron emission tomography scanning. An initial bronchoscopic exam revealed circumferential narrowing of the entire left main stem bronchus with necrotic and friable material on the medial wall. Neither aspirates from this necrotic area nor bronchial washing were diagnostic. A second bronchoscopy with endobronchial ultrasound evidenced a soft tissue thickening on the medial aspect of the left main stem bronchus underlying the area of necrosis visible endoluminally. Endobronchial ultrasound-guided transbronchial needle aspiration performed in this area revealed multiple fungal elements suggestive of *Aspergillus species*.

**Conclusion:**

We describe the first case of invasive aspergillus tracheobronchitis in which the diagnosis was facilitated by the use of endobronchial ultrasound guided trans-bronchial needle aspiration. To the best of our knowledge, we are also presenting the first positron emission tomography scan images of this condition in the literature. We cautiously suggest that endobronchial ultrasound imaging may be a useful tool to evaluate the degree of invasion and the involvement of vascular structures in these patients prior to bronchoscopic manipulation of the affected areas in an effort to avoid potentially fatal hemorrhage.

## Introduction

Invasive aspergillosis is one of the most common fungal infections in immunocompromised hosts, involving the respiratory tract in 90% of cases [1]. This disease occurs almost exclusively in immunosuppressed and especially myelosuppressed patients, although there have been rare patients without any grossly apparent immune defect. The most common form of aspergillus species infection in immunocompromised patients is invasive pulmonary aspergillosis, which mainly involves the lung parenchyma and, rarely, the trachebronchial tree [2]. Infection confined only to the tracheobronchial tree is known as invasive aspergillus tracheobronchitis (IATB), and it generally carries an ominous prognosis. The diagnosis of this condition is usually delayed due to its non-specific presentation. We are presenting a case of IATB in which the diagnosis was obtained by endobronchial ultrasound (EBUS)-guided fine needle aspiration (FNA) after initially failing to reach the diagnosis through flexible bronchoscopy. Additionally, although lesions caused by aspergillus in the lungs have already been shown to have increased fluorodeoxyglucose (FDG) activity on positron emission tomography (PET) scanning [3,4], we are presenting the first PET scan images of IATB in the literature.

## Case presentation

**Figure 1:**
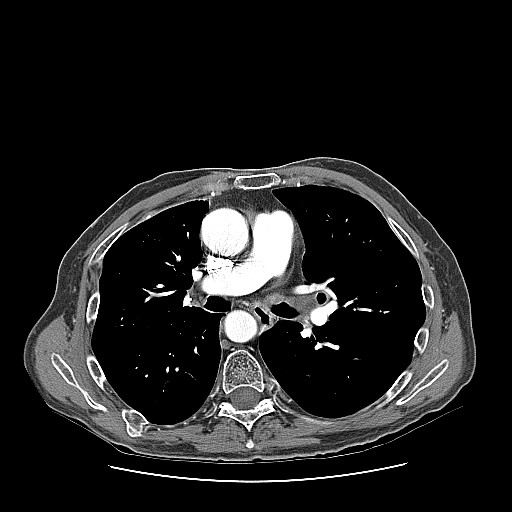
**Computer tomography (CT) of the chest showing soft tissue thickening around LMS bronchus**.

**Figure 2:**
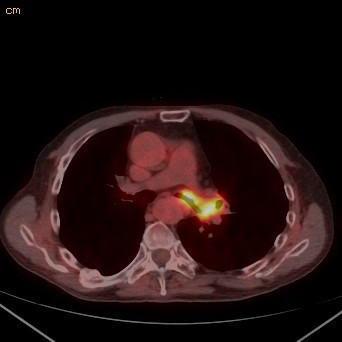
**PET-CT (positron emission tomography - computer tomography) scan showing FDG-avid circumferential thickening of distal LMS bronchus and left upper lobe (SUV of 7.4)**.

A 65-year-old Hispanic man from Bolivia with chronic lymphocytic leukemia (CLL) diagnosed 11 years before presentation and initially treated with alkylating agents, steroids and purine analogs, underwent an allogenic stem cell transplant from an HLA-compatible sibling a year before presentation. He developed cough and malaise eight months after the transplant for which he was admitted to another facility. Chest radiographs were reportedly negative and the patient was diagnosed with bronchitis and sent home on oral antibiotics. He continued to have intermittent cough productive of clear sputum associated with worsening malaise and weakness for about four months and was eventually evaluated at our hospital. Computed tomography (CT) of the chest revealed diffuse soft tissue thickening around the left main stem (LMS) bronchus (Figure 1). This area was also intensely FDG-avid on PET scanning with a maximum standardized uptake value (SUV) of 7.4 (Figure 2). The radiologic interpretation was consistent with leukemic infiltration of the bronchus or, less likely, localized graft-versus-host disease. Inflammation and mucosal thickening of the walls of the sphenoid sinuses were also noted incidentally on the same PET/CT scan and interpreted as sinusitis.

His medical history was remarkable for adult-onset asthma, two episodes of pneumonia several years ago, actinic keratosis and squamous cell carcinoma of the skin. He was a lifelong non-smoker and was on immunosuppression with prednisone and tacrolimus, and routine prophylaxis with atovaquone, valacyclovir and voriconazole. His vital signs were stable and his respiratory exam was unremarkable. He was thrombocytopenic (21,000/mm^3^), but not neutropenic (absolute neutrophil count 3,960/mm^3^).

**Figure 3:**
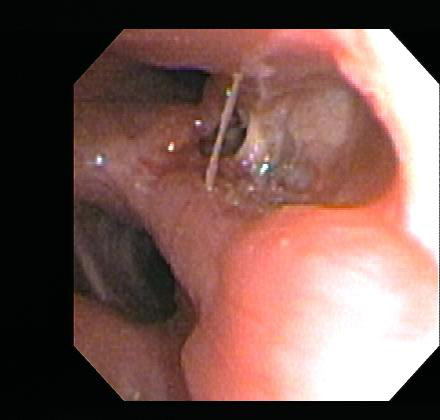
**Bronchoscopic exam corroborated the circumferential narrowing of the entire LMS (left main stem) bronchus**. The medial wall of the distal aspect of the LMS bronchus was ulcerated and covered with clear-coloured necrotic material.

**Figure 4:**
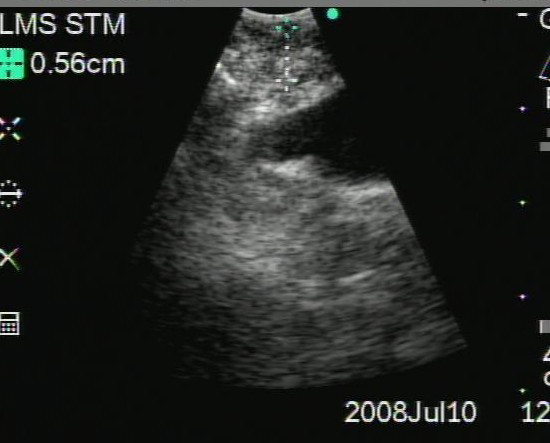
**Convex-probe EBUS (7.5 MHz, depth 4 cm) evidenced soft tissue thickening and a heterogenous and eliptical mass on the medial aspect of the LMS bronchus**.

**Figure 5:**
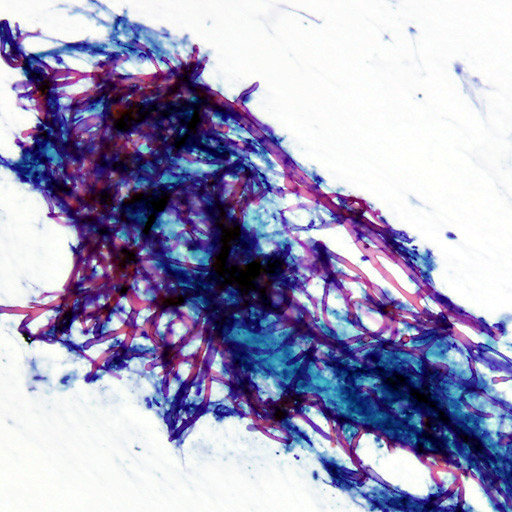
**On-site cytopathology evaluation revealed abundant necrotic material with multiple mycelia consisting of septate hyphae branching at about 45 degrees, characteristic of aspergillus species**.

An initial bronchoscopic exam revealed circumferential narrowing of the entire LMS bronchus (Figure 3). The mucosa on the medial wall of the distal end of the LMS bronchus appeared to be necrotic and friable. This necrotic area measured about 1.5 cm in diameter. The necrotic area was washed with normal saline but biopsies were not taken because significant superficial bleeding was noted during minimal suctioning. Cytologic analysis of the bronchial wash revealed fragments of cartilage without diagnostic features. Stains and cultures for bacteria, AFB and fungus were negative. The patient's thrombocytopenia was known to be refractory to platelet transfusions. In an effort to safely expedite a diagnosis, a second bronchoscopy was performed utilizing convex-probe EBUS (Olympus UC-160F, Olympus America Inc., Center Valley, PA) with the goal of performing directed biopsies while avoiding vascular structures. Under ultrasound examination, a thickening of soft tissue was noted on the medial aspect of the LMS bronchus, underlying the area of necrosis visible endoluminally (Figure 4). The thickening of the soft tissue on the anterior and lateral aspect of the distal LMS was also observed, with no evidence of a distinct lymph node in this location. With EBUS guidance, transbronchial needle aspiration (TBNA) biopsies were obtained from this abnormal region. On-site cytopathology evaluation revealed abundant necrotic material with multiple mycelia consisting of septate hyphae branching at around 45 degrees (Figure 5), characteristic of aspergillus species. The patient tolerated the procedure, which was free of complications. Based on our findings, the patient was immediately started on high-dose intravenous liposomal amphotericin B and posaconazole. He was discharged after ten days of therapy, but re-admitted seven days later with worsening respiratory symptoms and partial left upper lobe collapse. He was discharged again after two more weeks of intravenous antifungals with clinical and radiographical improvement.

## Discussion

Human aspergillosis can be classically divided as invasive, saprophytic or allergic. *Aspergillus fumigatus *is the species most commonly responsible for invasive aspergillosis, followed by *Aspergillus flavus*, *Aspergillus niger *and *Aspergillus terreus *[5]. These fungi can use the lower respiratory tract, sinuses or skin as entry portals to cause invasive infections. Inhalation of airborne aspergillus spores results in colonization of the respiratory mucosal surfaces. The progression from colonization to tissue invasion and the type of disease that patients develop depend mainly on their immune status and on local defense mechanisms [6,7]. The most common form of invasive aspergillosis in immunocompromised patients is invasive pulmonary aspergillosis. IATB is a rare manifestation defined as localized invasion of the bronchial wall by aspergillus. Young *et al*. reviewed the postmortem findings in 98 cases of aspergillosis and found that the infection was limited to the tracheobronchial tree in only five patients [2].

Three morpholgical variants of IATB have been described: obstructive tracheobronchitis, ulcerative tracheobronchitis and pseudomembranous necrotizing bronchial aspergillosis (PNBA) [6,8,9]. The obstructive form is characterized by massive intraluminal growth of aspergillus species associated with thick mucus plugs that generally produce atelectasis. Ulcerative lesions like the one we found in our patient penetrate through the tracheo-bronchial wall, and can create bronchoesophageal or bronchoarterial fistulas that may produce fatal hemorrhage [9,10]. In fact, Putnam *et al*. [10] reported a case of IATB localized to the right main stem bronchus and invading the right pulmonary artery in which the patient had a fatal hemorrhage after bronchoscopic manipulation. PNBA is characterized by extensive formation of whitish pseudomembranes composed of hyphae, fibrin and necrotic debris. Rather than three distinct entities, these morphologic variants may just represent different stages in the development of IATB [6,8].

The clinical mainfestations of IATB are entirely different from those of invasive pulmonary aspergillosis. The insidious presentation with non-specific symptoms and the paucity of findings in chest roentgenograms often delay the diagnosis, giving this disease an ominous prognosis [10-12]. Airway-related symptoms such as cough, dyspnea, wheezing and hemoptysis are cardinal features. There is little documentation of the radiologic features of IATB in the literature. As previously mentioned, lesions caused by aspergillus in the lungs and other organs are known to have increased FDG activity on PET scanning [3,4]. Nevertheless, to the best of our knowledge, we are presenting the first PET/CT scan images of IATB in the literature (Figure 2).

The diagnosis of IATB is almost always confirmed by bronchoscopic examination and sampling. Although thrombocytopenia is commonly a limiting factor for acquiring endobronchial biopsies, bronchoscopic aspiration of debris and bronchial washings allow diagnosis in the majority of cases by showing the presence of *Aspergillus *hyphae in special stains or by recovering the organism in fungal cultures. The samples obtained during the first bronchoscopic examination of our patient, however, were negative. As the patient's thrombocytopenia was refractory to platelet transfusions, we opted for EBUS-guided TBNA in order to achieve diagnosis with the lowest risk of bleeding. This is the first case of IATB in which diagnosis was facilitated by EBUS that we could find in the medical literature. It is our opinion that real-time EBUS might also be useful in delineating the relationships during fungal invasion into adjacent tissues and the involvement of major vascular structures, potentially preventing lethal hemorrhage. This type of infection can progress very rapidly, leading to invasion of major vessels in the time between CT imaging and bronchoscopy, making routine bronchoscopic manipulation and sampling of debris extremely dangerous and even fatal [10].

## Conclusion

In conclusion, IATB is a rare form of invasive aspergillosis affecting mainly immunocompromised patients. The non-specific clinical presentation often leads to late diagnosis and poor prognosis. We report the first case of IATB diagnosed by EBUS-guided TBNA. We also cautiously suggest that EBUS imaging may be a useful tool to evaluate the depth of fungal invasion into adjacent tissues and the involvement of vascular structures in these patients prior to bronchoscopic manipulation of the affected areas in an effort to avoid fatal hemorrhage.

## Consent

Written informed consent was obtained from the patient for publication of this case report and accompanying images. A copy of the written consent is available for review by the journal's Editor-in-Chief.

## Competing interests

The authors declare that they have no competing interests.

## Authors' contributions

RA performed initial bronchoscopic exam. RFC, CAJ, MS, RM and GAE performed EBUS. All authors equally contributed to the creation of the manuscript.

## References

[B1] Meyer RD Rosen P Armstrong D Yu B Aspergillosis complicating neoplastic disease Am J Med 1973 54 6 15 10.1016/0002-9343(73)90077-6 4345262

[B2] Young RC Bennett JE Vogel CL Carbone PP DeVita VT Aspergillosis, the spectrum of the disease in 98 patients Medicine 1970 49 147 173 10.1097/00005792-197003000-00002 4913991

[B3] Sonet A Graux C Nollevaux MC Krug B Bosly A Borght T Vander Unsuspected FDG-PET findings in the follow-up of patients with lymphoma Ann Hematol 2007 86 9 15 10.1007/s00277-006-0167-4 17021839

[B4] Wilkinson MD Fulham MJ McCaughan BC Constable CJ Invasive aspergillosis mimicking stage IIIA non-small-cell lung cancer on FDG positron emission tomography Clin Nucl Med 2003 28 234 235 10.1097/00003072-200303000-00017 12592137

[B5] Barnes PD Marr KA Aspergillosis: spectrum of disease, diagnosis and treatment Infect Dis Clin North Am 2006 20 545 561 10.1016/j.idc.2006.06.001 16984868

[B6] Kramer MR Denning DW Marshall SE Ross DJ Berry G Lewiston NJ Stevens DA Theodore J Ulcerative tracheobronchitis after lung transplantation. A new form of invasive aspergillosis Am Rev Respir Dis 1991 144 552 556 1654038 10.1164/ajrccm/144.3_Pt_1.552

[B7] Clark A Skelton J Fraser RS Fungal tracheobronchitis. Report of 9 cases and review of the literature Medicine 1981 70 1 14 10.1097/00005792-199101000-00001 1988763

[B8] Denning DW Commentary: unusual manifestations of aspergillosis Thorax 1995 50 812 813 10.1136/thx.50.7.812 7570425 PMC474663

[B9] Patel N Talwar A Stanek A Epstein M Tracheobronchial Pseudomembrane Secondary to Aspergillosis J Bronchol 2006 13 147 150 10.1097/00128594-200607000-00011

[B10] Putnam J Dignani M Mehra R Anaissie E Morice R Libshitz H Acute Airway Obstruction and Necrotizing Tracheobronchitis from Invasive Mycosis Chest 1994 106 1265 1267 10.1378/chest.106.4.1265 7924508

[B11] Machida U Kami M Kanda Y Takeuchi K Akahane M Yamaguchi I Kakiuchi C Takeda N Tanaka Y Chiba S Honda H Hirai H Aspergillus tracheobronchitis after allogenic bone marrow transplantation Bone Marrow Transplant 1999 24 1145 1149 10.1038/sj.bmt.1702030 10578166

[B12] Sayiner A Kürşat S Töz H Duman S Onal B Tümbay E Pseudomembranous necrotizing bronchial aspergillosis in a renal transplant recipient Nephrol Dial Transplant 1999 14 1784 1785 10.1093/ndt/14.7.1784 10435898

